# Dexamethasone induced inhibition of Dectin-1 activation of antigen presenting cells is mediated via STAT-3 and NF-κB signaling pathways

**DOI:** 10.1038/s41598-017-04558-z

**Published:** 2017-07-03

**Authors:** Philipp Kotthoff, Annkristin Heine, Stefanie Andrea Erika Held, Peter Brossart

**Affiliations:** Medical Clinic III for Oncology, Hematology, Immuno-Oncology and Rheumatology, University Hospital Bonn Sigmund-Freud-Straße 25, 53127 Bonn, Germany

## Abstract

Treatment of patients with glucocorticoids can result in an increased risk of infection with pathogens such as fungi. Dectin-1 is a member of the C-type lectin receptor superfamily and was shown to be one of the major receptors for fungal beta-glucans. Activation of Dectin-1 increases the production of cytokines and chemokines and T-cell stimulatory capacity of DC and mediates resolution of fungal infections. Here we show that antigen-presenting cells generated in the presence of dexamethasone (Dex-DC) have a reduced capacity to stimulate T-cell proliferation and decreased expression of costimulatory molecules, that can not be enhanced upon stimulation with Dectin-1 ligands. Stimulation of Dex-DC with beta-glucans induced a strong upregulation of Syk phosphorylation and increased secretion of IL-10, while the production of IL-12, IL-23 and TNF-alpha was reduced. Downstream of Syk stimulation of Dectin-1 on Dex-DC resulted in phosphorylation of STAT3 and reduced nuclear localization of transcription factors involved in DC activation and function.

## Introduction

Dexamethasone is a widely used synthetic glucocorticoid that suppresses the immune system. Long-term treatment with dexamethasone often results in increasesd susceptibility to infections. Among other pathogens, *candida albicans* is a major threat to immune compromised individuals^1^. Treatment with dexamethasone impairs the maturation of DC *in vivo* and *in vitro*. Dexamethasone treated dendritic cells (Dex-DC) show a tolerogenic phenotype which is characterized by lack of co-stimulatory molecules and inhibition of T-cell proliferation^2–4^. *In vitro*, dexamethasone treatment of DC during their differentiation results in a durable state of immaturity upon stimulation with different ligands^3, 4^.

Dectin-1 is one of the major beta-glucan receptors on DC, macrophages and neutrophils^5, 6^. Beta-Glucans are polysaccharides connected by 1-3-β -glycosidic bonds and represent the major component of the fungal cell wall^7^. Stimulation of Dectin-1 by beta-glucans induces a variety of immune stimulatory effects, which mediate innate and adaptive immune responses. Among innate mechanisms, Dectin-1 facilitates phagocytosis, production of reactive oxygen species (ROS) and the release of a large variety of cytokines including IL-10, IL-1beta, IL-6, IL-2, IL-23, IL-12, IFN-beta and TNF-alpha^8–11^. The adaptive response can be shaped by the release of distinct cytokines which lead to Th1/Th17-polarization of T-cells^12–14^. In addition, Dectin-1 was shown to activate cytotoxic CD8 T-cells and B-cells^15–17^. This plethora of immunologic effects can be induced by Dectin-1 alone or in collaboration with other pattern recognition receptors (PRR) such as toll like receptor (TLR)-2^13^. Downstream of Dectin-1, signaling is mainly transduced by the kinase Syk^17, 18^. Via CARD9 Dectin-1 is able to activate the inflammasome and to subsequently induce expression and processing of IL-1beta^7, 19^. Gringhuis *et al*. showed that signaling via Dectin-1 also activates a second Raf-1-dependent pathway, which integrates into the Syk-dependent pathway to modulate NF-kappaB singaling^20^.

Defects in the Dectin-1 molecule can result at least in an increased risk for mucosal infections with fungi in affected individuals^21^. In our study we analyzed Dectin-1 expression and signaling in Dex-DC and compared it to immature dendritic cells (iDC).

## Results

### Dendritic cells generated in the presence of dexamethasone express high levels of Dectin-1, but do not increase expression of maturation markers upon its stimulation

**Figure 1:**
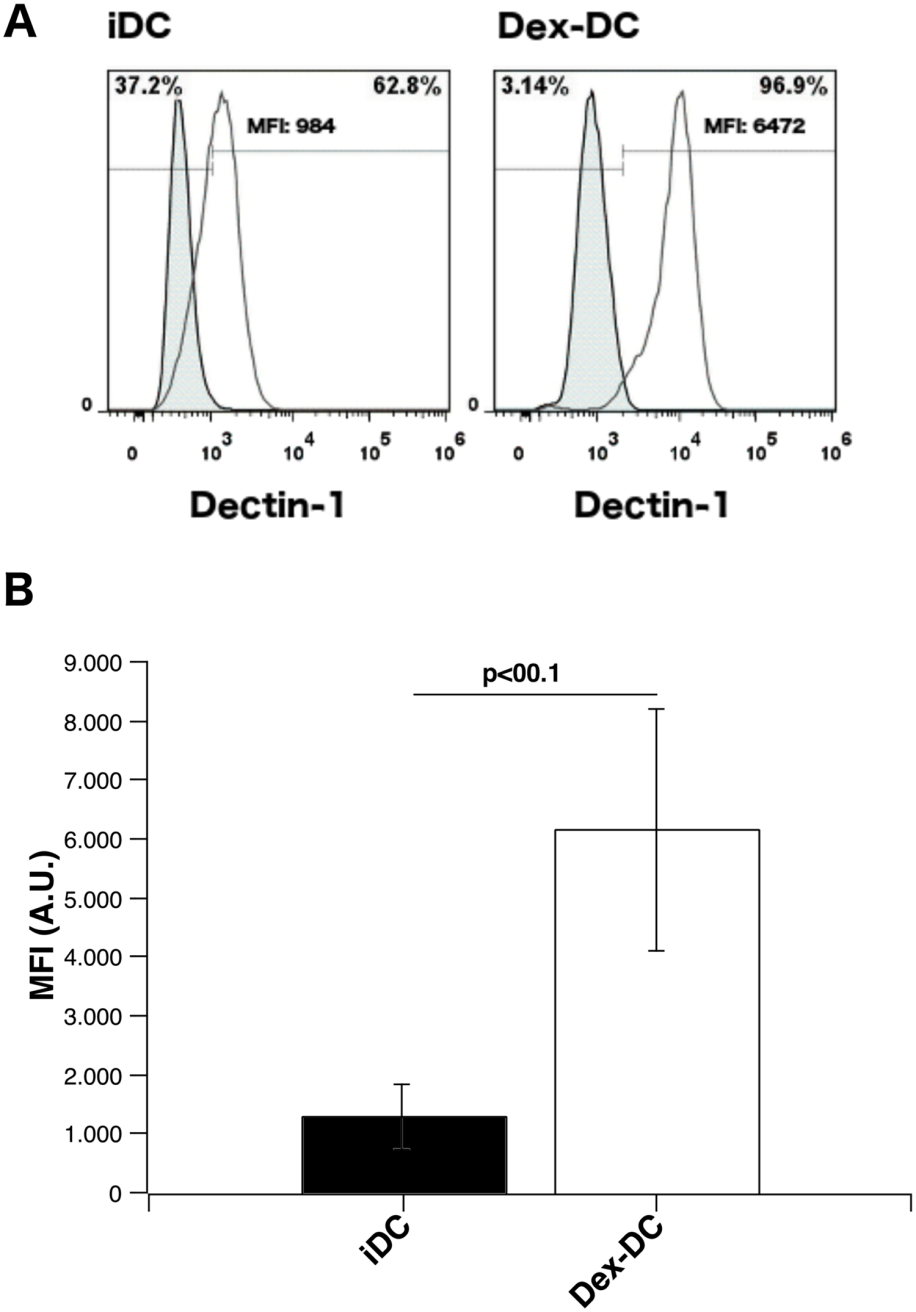
Dectin-1 expression is increased on the surface of dexamethasone-treated dendritic cells (Dex-DC) as compared to immature dendritic cells (iDC). (**A**) Representative histogram of Dectin-1 expression on the surface of iDC and Dex-DC. The tinted histogram represents the isotype control. (**B**) Pooled expression of Dectin-1 on iDC and Dex-DC from n = 7 independent experiments. Data are represented as mean ± SD. Unpaired t test was used to compare expression on iDC and Dex-DC. MFI of the isotype control was subtracted from cells stained with anti-Dectin-1-PE antibody (R&D Systems; FAB1859P).

**Figure 2:**
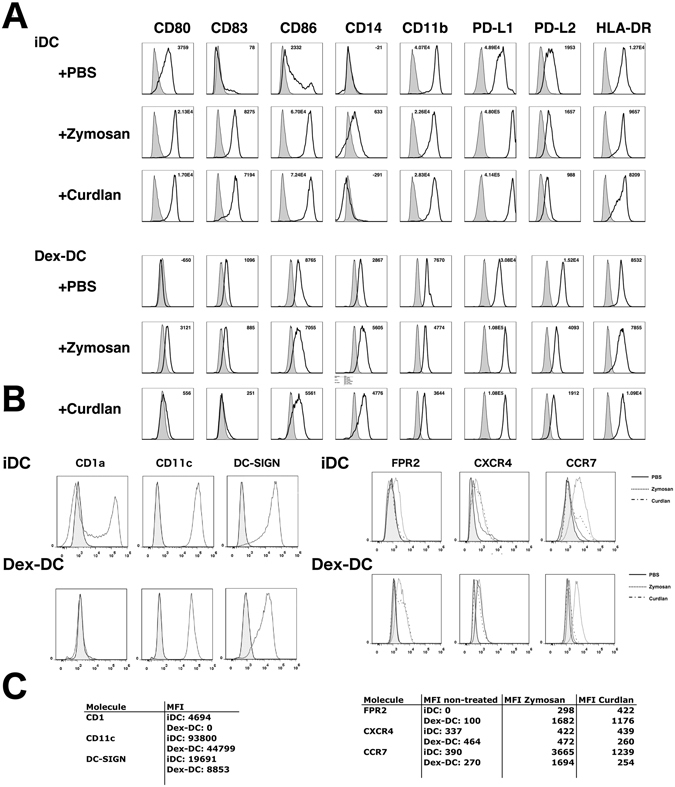
Dex-DC do not upregulate the expression of co-stimulatory molecules CD80, CD83 and CD86 upon stimulation with beta-glucans (**A**) iDC and Dex-DC were stimulated with 100 µg/ml zymosan or curdlan for 18 hours. Expression of surface markers was analyzed by flow cytometry. Representative histograms are shown out of three experiments. Tinted histograms represent isotype controls. (**B**) Left panel shows expression of the surface markers CD1a, CD11c and DC-SIGN. Isotype control is shown in grey. Right panel: Expression of chemokine receptors FPR2, CXCR4 and CCR7 on iDC or Dex-DC. Solid line shows expression after no treatment. Dotted land dashed ine show iDC or Dex-DC after treatmend with 100 µg/ml zymosan or curdlan respectively for 18 hours. (**C**) Quantification of MFI from histograms in (**B**).

Dectin-1 activation by beta-glucans mediates anti-fungal immunity and thereby balancing pro- and anti-inflammatory responses upon candida colonization^15, 19, 22^. First, we analyzed whether DC that were generated in the presence of dexamethason (Dex-DC) express Dectin-1 on their surface. We found that Dectin-1 was highly expressed on Dex-DC cells compared to iDC (Fig. 1A,B). In addition stimulation of iDC with 100 µg/ml zymosan or curdlan increased the expression of the maturation markers CD80, CD83 and CD86. In contrast, Dex-DC show a reduced upregulation of these markers (Fig. 2A). PD-L1 expression was found to be increased on both cell subsets after Dectin-1 stimulation with zymosan or curdlan. PD-L2 and HLA-DR were expressed at lower levels on Dex-DC and iDC after stimulation with the indicated beta-glucans. CD11b was expressed at lower levels on Dex-DC as compared to iDC and was downregulated on both cell subsets after stimulation with beta-glucans. CD11b is part of CR3, which was found to be another beta-glucan receptor and collaborating receptor of Dectin-1^23^.

Further characterization of Dex-DC showed that these cells lack CD1a expression and remain CD14 positive as described previously^4^. DC-SIGN, another receptor for *C. albicans* uptake^24^, was also expressed at lower levels on Dex-DC (Fig. 2B,C). Chemokine receptors like CXCR4, CCR7 and FPR2 were expressed on both subsets, presumably enabling both types of antigen-presenting cells to migrate along chemokine gradients to the lymph nodes (Fig. 2B,C). The expression of ox40-L was low on both cell types, and was not affected by treatment with dexamethasone and curdlan (Figure S2). In immature dendritic cells generated in the presence of IL-4 and GM-CSF the addition of curdlan resulted in an upregulation of ox40 L on the cell surface.

### Dectin-1 stimulated Dex-DC lack allogeneic T-cell stimulatory capacities

Activation of T-cells requires their interaction with antigen-presenting cells. Apart from interaction of the T-cell-receptor with MHC-I/II molecules (*signal one*), a second signal (*signal two*) is required which is facilitated by the interaction with co-stimulatory molecules. A third signal (*signal three*) is characterized by secretion of cytokines, which polarize T-cells to distinct subsets. Absence of signal two anergizes T-cells.

**Figure 3:**
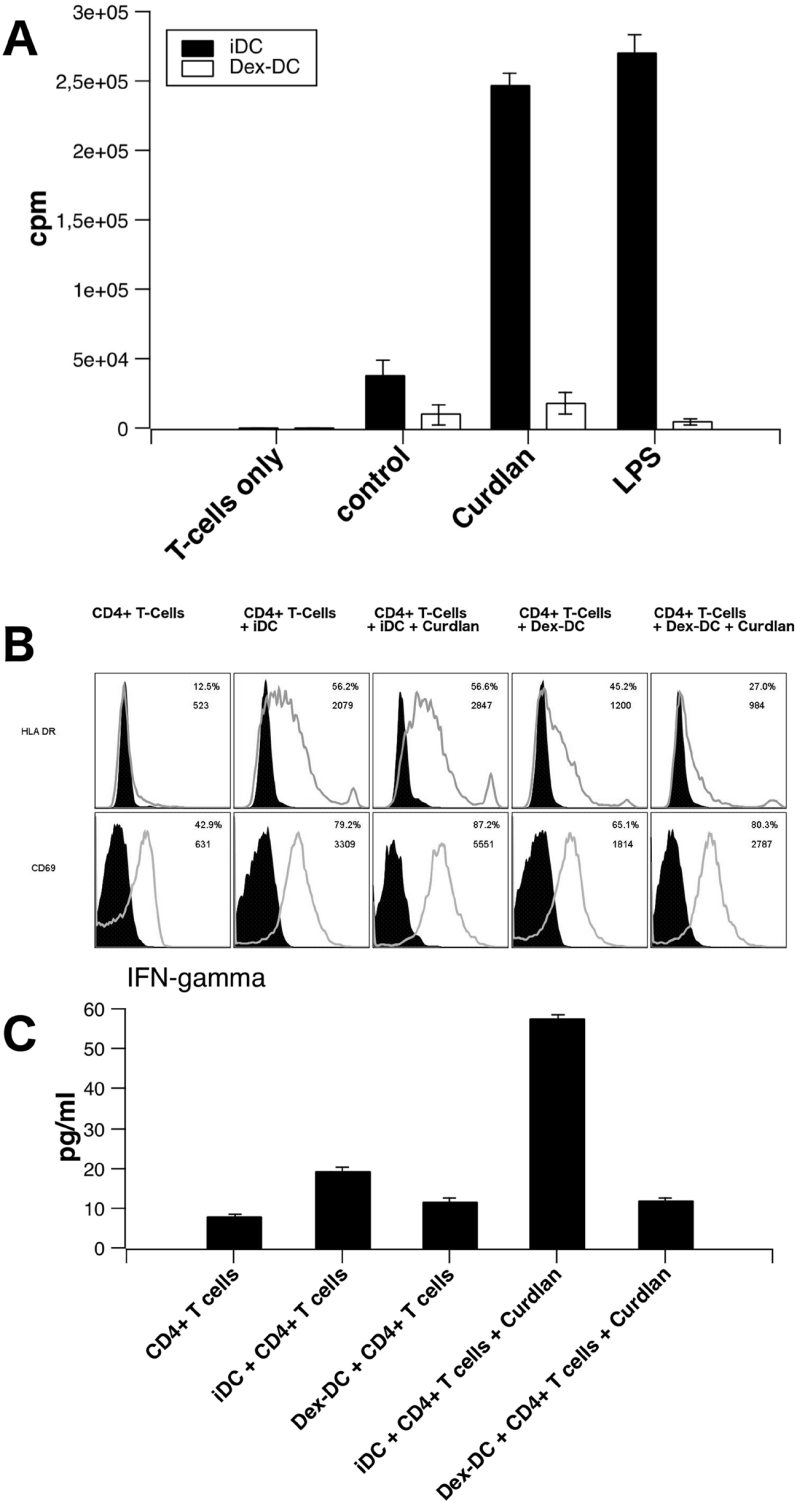
Dex-DC do not induce proliferation of allogeneic T-cells after stimulation with curdlan or LPS or induce expression of T-Cell maturation markers CD69 and HLA-DR and IFN-γ. (**A**) Untreated or beta-glucan stimulated (zymosan or curdlan: 100 µg/ml) iDC or Dex-DC were seeded at 1 × 10^4^ per well and incubated together with 1 × 10^5^ CD4^+^ t-cells for 5 days. At day 5, [^3^H]-Thymidin was added to the co-cultured cells for 16 h. Proliferation of allogeneic t-cells was analyzed by counting decay of incorporated [^3^H]-Thymidin. Data are presented as mean ± SD. (**B**) Allogeneic CD4^+^ T-Cells were purified, incubated with iDC or Dex-DC that were either untreated or stimulated with 100 µg/ml curdlan. Expression of CD69 and HLA-DR on CD4^+^ T-cells was assessed by flow-cytometry. One representative experiment is shown. MFI and percentage of positive cells is indicated. (**C**) Supernatants of cocultured CD4^+^ T-cells were analyzed for IFN-gamma secretion as described.

Dexamethasone-treatment of DC resulted in reduced capacity to stimulate the proliferation of allogeneic T-cells in a mixed lymphocyte reaction (MLR) assay that could not be increased by stimulation with Dectin-1 ligand curdlan or the TLR-4 ligand LPS (Fig. 3A).

To further analyze the effects of Dex-DC on T cells we assessed the expression of activation markers such as HLA-DR and CD69 on CD4^+^ T-cells incubated with iDC or Dex-DC. Figure 3B shows the surface expression of both markers on allogeneic CD4^+^ T-cells incubated with unstimulated or curdlan activated iDC or Dex-DC. Both maturation markers were expressed at lower levels on T-cells incubated with Dex-DCs. Furthermore, these CD4^+^ allogeneic T-Cells secreted lower levels of IFN-gamma in the presence of Dex-DCs (Fig. 3C).

### Cytokine secretion of Dex-DC upon stimulation with curdlan

Next we assessed the cytokine secretion of Dex-DC after stimulation with 100 µg/ml curdlan. Curdlan is a more specific ligand for Dectin-1 that in contrast to zymosan has lower TLR -stimulating properties^25^.

**Figure 4:**
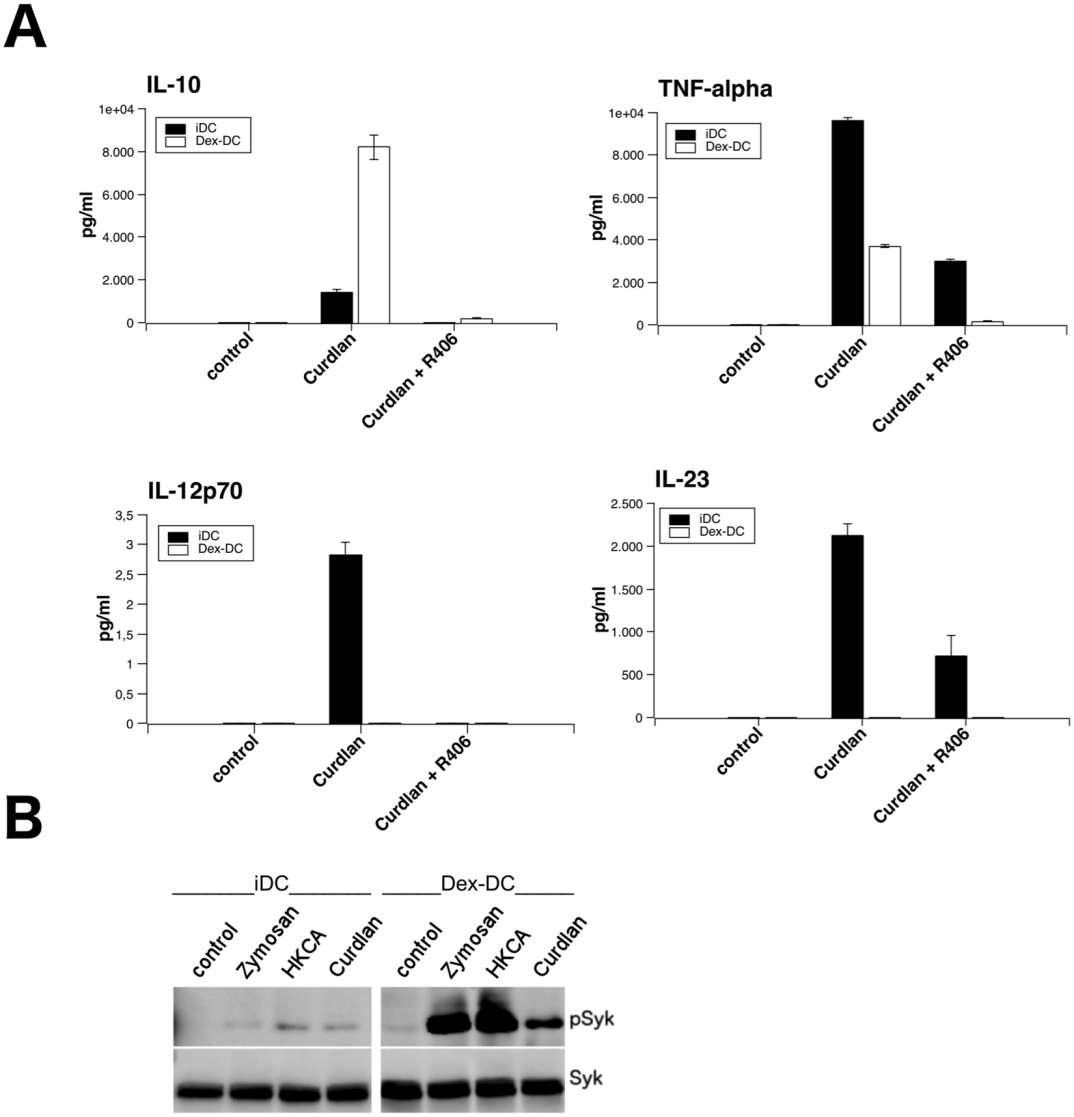
Cytokine production and Syk phosphorylation of Dex-DC and iDC upon stimulation with Dectin-1 ligands. (**A**) Secretion of cytokines by untreated or curdlan stimulated iDC or Dex-DC. Supernatants of iDC and Dex-DC were used in a sandwich-ELISA to determine the concentration of secreted cytokines. Data are presented as mean ± SD of one out of three experiments. (**A**) Secretion of IL-10, TNF-alpha, IL-12p70 and IL-23 after stimulation of iDC or Dex-DC with 100 µg/ml curdlan. (**B**) Western-Blot analysis of Syk-phosphorylation at tyrosines 525 and 526. Lysates of beta-glucan stimulated (zymosan or curdlan: 100 µg/ml; 1 × 10^7^ HKCA; 30 min) iDC and Dex-DC were separated by SDS-PAGE and proteins were transferred to nitro-cellulosis, blocked and analyzed for phosphorylated Syk. For clarity lanes were cropped from a single immunoblot.

DC generated in the presence of dexamethasone secreted lower amounts of TNF-alpha, IL-23 and IL-12p70 upon stimulation with Curdlan (Fig. 4). Interestingly, secretion of IL-10 was increased in supernatants of Dex-DC. It was previously shown that the secretion of cytokines by DC after stimulation with Dectin-1 is Syk dependent. In line with this we show that an inhibitor of Syk, R406, reduced the production of cytokines by iDC and Dex DC upon incubation of these cells with the Syk-inhibitor R406 (2.5 µM) prior to Dectin-1 stimulation with curdlan (Fig. 4A).

In the next set of experiments we analyzed the phoshorylation of Syk in iDC and Dex-DC after stimulation for 30 min with different Dectin-1 ligands (Fig. 4B) such as HKCA, Zymosan and Curdlan. Stimulation of Dectin-1 in Dex-DC resulted in increased Syk-phosphorlyation as compared to iDC.

### Syk hyperactivation in Dex-DC results in increased generation of superoxide-anions

**Figure 5:**
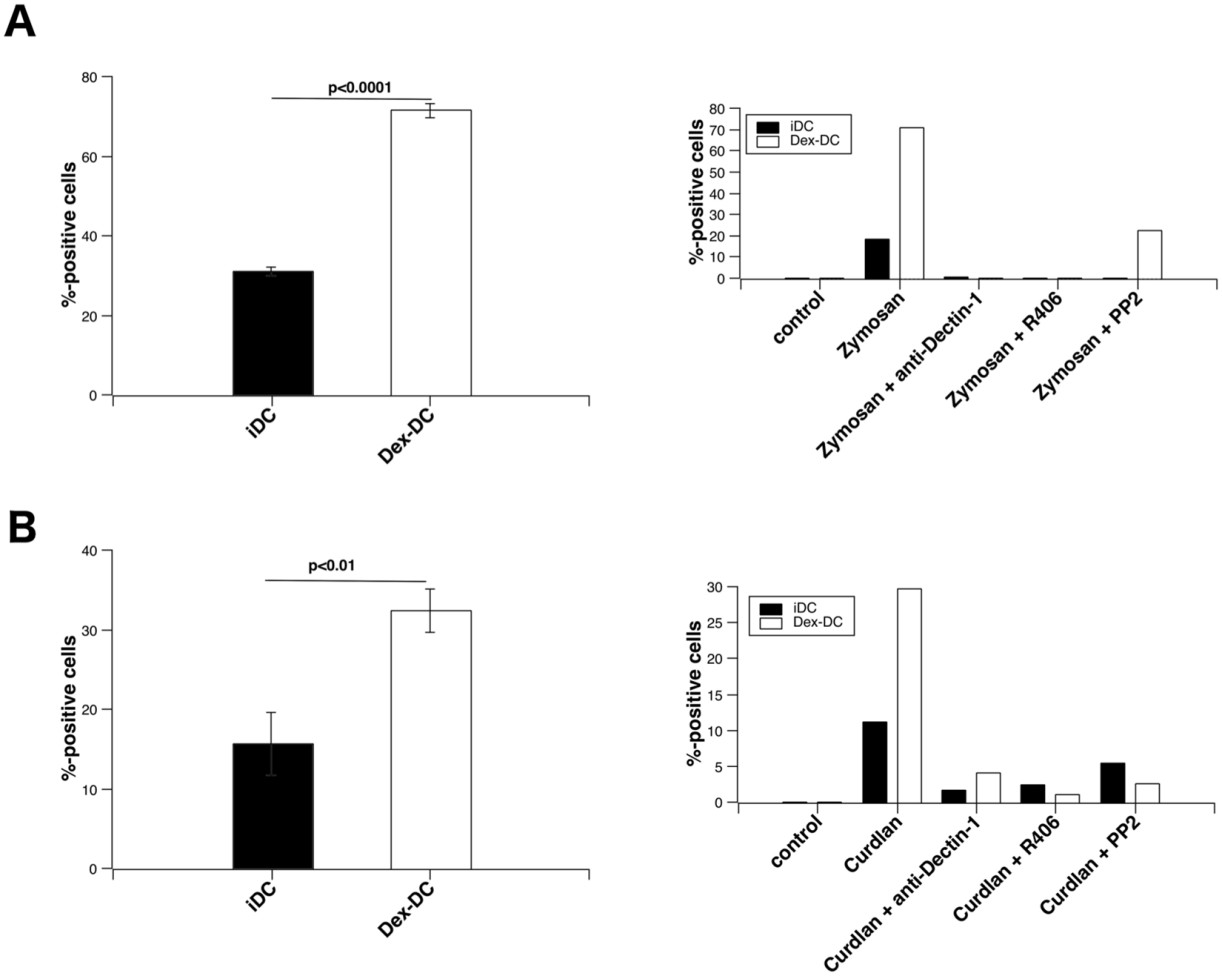
Production of superoxide-anions is increased in Dex-DC and depends on Dectin-1, Syk and src. (**A**,**B**) Generation of superoxide-anions was analyzed in iDC and Dex-DC after stimulation with zymosan (100 µg/ml) or curdlan (400 µg/ml). (**A**) Left panel: Superoxide-anion production in iDC and Dex-DC after zymosan-stimulation. Data are represented as mean ± SD from three independent experiments. Unpaired t-test was used to compare iDC and Dex-DC. Right panel: Superoxide-anion production of iDC and Dex-DC after Syk/src-inhibition and Dectin-1 blockade. Inhibitors were added to the cells 30 min prior to stimulation with zymosan. R406 was used at 2.5 µM and PP2 at 5 µM. Anti-Dectin-1 antibody was used at 5 µg/ml. (**B**) Left panel: Superoxide-anion production in iDC and Dex-DC after curdlan-stimulation. Data are represented as mean ± SD from three independent experiments. Unpaired t-test was used to compare iDC and Dex-DC. Right panel: Superoxide-anion production of iDC and Dex-DC after Syk/src-inhibition and Dectin-1 blockade. Inhibitors were added to the cells 30 min prior to stimulation with zymosan. R406 was used at 2.5 µM and PP2 at 5 µM. Anti-Dectin-1 antibody was used at 5 µg/ml.

Syk-mediated signaling is involved in the generation of ROS in macrophages^18^, DC and neutrophils^26^. Dectin-1 stimulation was previously reported to induce superoxide-anion production^18, 27^. We found that compared to iDC the production of superoxide-anions was highly increased in zymosan or curdlan stimulated Dex-DC (Fig. 5), which was dependent on src, Syk and Dectin-1. Curdlan induced lower amounts of superoxide-anions in Dex-DC and iDC within 45 min of stimulation and higher concentrations of curdlan were required to induce superoxide-anion generation (also see Figure S1).

### Dectin-1 stimulation results in activation of the MAP-kinases Erk, p38 and STAT3 in Dex-DC

**Figure 6:**
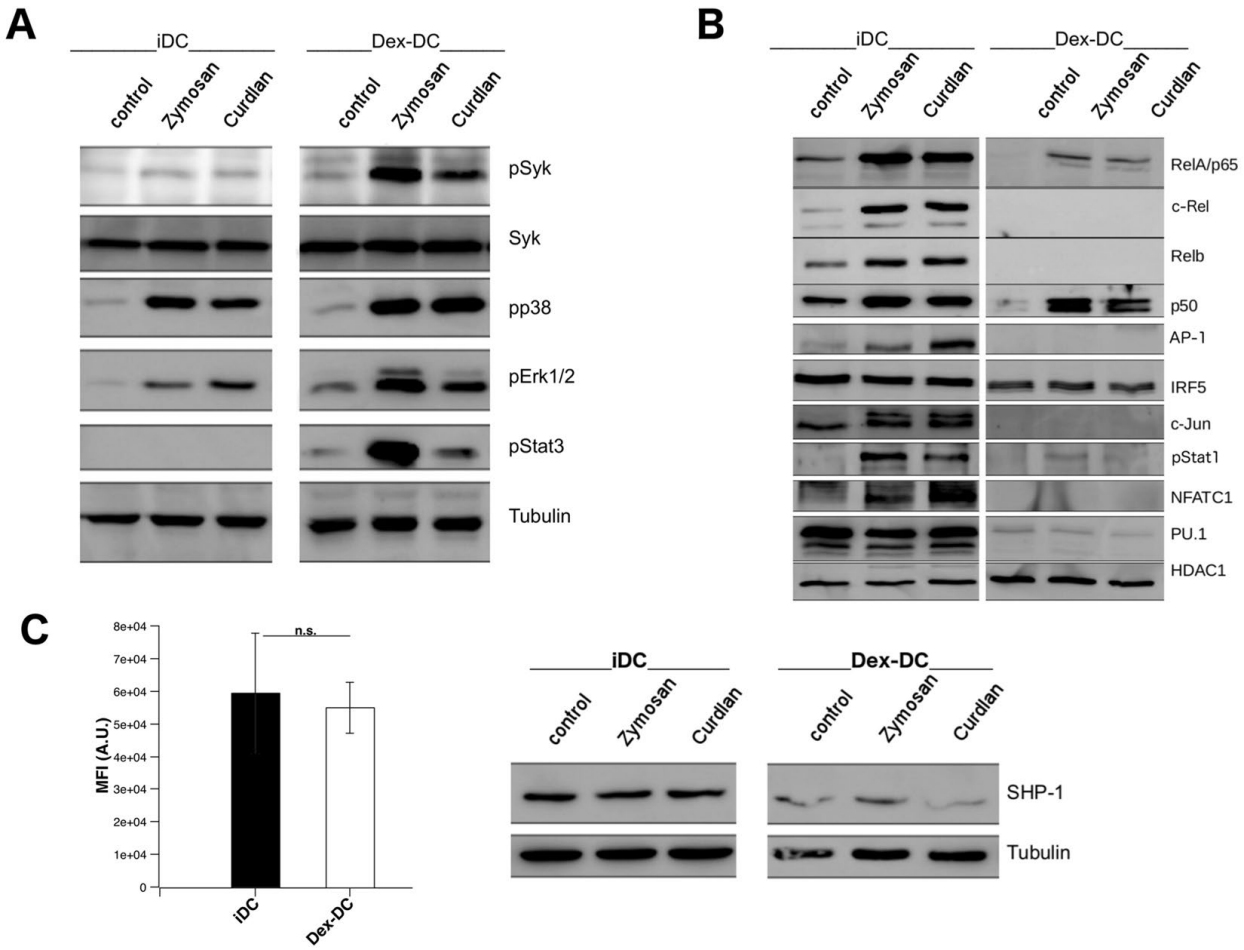
Signal transduction downstream of Syk is altered in Dex-DC upon stimulation with zymosan or curdlan and may be mediated by SHP-1. (**A**) Lysates of untreated, zymosan- or curdlan-treated (100 µg/ml; 30 min) were separated by SDS-PAGE and transferred to nitro-cellulosis, blocked and analyzed for phosphorylated p38, Erk1/2 and Stat3. Tubulin was used as a loading control. For clarity lanes were cropped from a single immunoblot. (**B**) Nuclear lysates of untreated, zymosan- or curdlan-treated (100 µg/ml; 4 h) were separated by SDS-PAGE and transferred to nitro-cellulosis, blocked and analyzed for translocation of the indicated proteins. HDAC1 was used as a loading control. For clarity lanes were cropped from a single immunoblot. (**C**) left panel: Expression of CD45 on the surface of iDC and Dex-DC. Cells were harvested and stained with anti-CD45 antibody or appropriate isotype control antibody. Data are represented as mean ± SD from eight experiments. Right panel: iDC and Dex-DC were left untreated or were treated with zymosan or curdlan (100 µg/ml; 30 min). Proteins of cell lysates were separated by SDS-PAGE and transferred to nitro-cellulosis, blocked and analyzed for SHP-1 expression. Tubulin was used as a loading control. For clarity lanes were cropped from a single immunoblot.

Downstream of Syk, stimulation of Dectin-1 induces the activation of MAP-Kinases via CARD9^28^. As shown in Fig. 6A stimulation of Dex-DC with beta-glucans resulted in increased phosphorylation of Erk and p38. Interestingly, STAT3 phosphorylation was only detected in Dex-DC upon stimulation with beta-glucans. STAT3 mediates immune inhibitory effects on DC function by promoting expression and secretion of anti-inflammatory cytokines like IL-10^29^ and subsequent inhibition of Th1- and Th17-mediated response^29, 30^. Phosphorylation of STAT3, Erk and p38 was decreased in lysates of Dex-DCs when pretreated with the Syk-inhibitor R406 (5 µM) or an anti-Dectin-1 antibody (10 µg/ml) (Figure S3).

### Translocation of the transcription factors NF-κB, NFAT, PU.1, AP.1 and c-Jun is inhibited in Dex-DC upon Dectin-1 stimulation

Next we assessed translocation of transcription factors involved in Dectin-1 activation and cytokine secretion to the nuclei of iDC and Dex-DC after incubation with beta-glucans (Fig. 6B). RelA, c-Rel and RelB, transcription factors of the NF-κB-family, were found at high levels in nuclear extracts of iDC after incubation with curdlan or zymosan whereas extracts of Dex-DC showed only low levels of RelA. Another member of the NF-κB-family, p50, was found to be upregulated in nuclei of iDC and Dex-DC after beta-glucan stimulation. p50 acts as a heterodimer together with p65 do induce transcription. p50-homodimers act as repressors of transcription of pro-inflammatory cytokines^31^, but may induce IL-10 production^32, 33^. NFATC1, PU.1, AP-1 and c-Jun were not detected in nuclear lysates of Dex-DC. STAT proteins are known to function as transcription factors. Upon phosphorylation, STAT-proteins translocate to the nucleus and induce trancription. Phosphorylated STAT1 was present in nuclear extracts of iDC after activation of Dectin-1 while it was highly reduced in nuclei of Dex-DC.

### The phosphatase SHP-1 might contribute to Syk-hyperactivation in Dex-DC

Syk-phosphorylation is dependent on the activity of membrane-proximal phosphatases. It was shown that the phosphatase CD45 dephosphorylates Syk after completion of Dectin-1-mediated engulfment^34^. We analyzed the expression of CD45 on the surface of iDC and Dex-DC by flow cytometry. We found that CD45 was expressed at similar levels on both cell types (Fig. 6C, left panel). Next we assessed the expression of SHP-1, which was shown to be involved in regulation of Syk- and STAT3-phosphorylation^35–37^. In our experiments expression of SHP-1 was reduced in Dex-DC (Fig. 6C, right panel), which might contribute to increased Syk-phosphorylation in Dex-DC.

## Discussion

Treatment with immunosuppressive agents such as dexamethasone increases the risk of fungal infections. One major threat is the infection with the commensal *C. albicans*, which can result in mucosal, but also severe systemic infections.

*C. albicans* is commonly found on healthy individuals where it is kept under control by innate mechanisms as well as Th1- and Th17-cells^38^. Dectin-1 is a non-Toll-like pattern recognition receptor and one of the major receptors for beta-glucans, which represent the main component of the fungal cell wall^5^. Dectin-1 is able to trigger innate and adaptive mechanism of the immune system. In humans, Dectin-1 is widely expressed on DC, macrophages, monocytes, neutrophils, eosinophils, B-cells and distinct T-cell subsets. Dectin-1 is able to induce the secretion of a variety of cytokines and chemokines and can either induce signaling alone or collaborate with Toll-like receptors or other C-type lectins e.g. CR3^23^. Downstream of Dectin-1, signaling is mainly mediated by Syk.

Here we show that dexamethasone treated dendritic cells (Dex-DC) express high levels of Dectin-1 on their surface. In addition, a single stimulation of Dectin-1 with beta-glucans leads to upregulation of Syk phosphorylation at tyrosine residues 525/526 as compared to iDC. Downstream of Syk, we observed a highly increased activation of STAT3 signaling while the induction of STAT3 signaling was absent in iDC. In the nuclei of Dex-DC, we found a reduced expression of NF-κB members RelB, c-Rel and RelA and other transcription factors such as AP-1, c-Jun, NFATC1, STAT1 and PU.1 upon stimulation of Dectin-1 with zymosan or curdlan. NF-κB-family transcription factors are important for differentiation and activation of DC^39^ and secretion of cytokines like IL-12 and subsequent Th-1 polarization^40^. The transcription factor PU.1 regulates DC development^41^. NFAT is a transcription factor that is induced by Dectin-1 mediated signaling and promotes cytokine secretion and activation of transcription factors in macrophages and dendritic cells^9^. AP-1 and c-Jun play a role in DC maturation^42^ and inflammatory responses^43^. Interestingly, p50 was the only member of the NF-κB family we were able to detect in nuclei of Dex-DC treated with beta-glucans. p50 homodimers are thought to be involved in production of IL-10^32^. These results were in line with STAT3 activation and increased secretion of IL-10. The importance of Syk activation in connection with Dectin-1 mediated IL-10 secretion was already shown by Rogers *et al*.^11^. They have shown that Syk is essential for Dectin-1mediated IL-10 but not IL-12 secretion in BMDC. The observed increased generation of superoxide-anions in our experiments by Dex-DC upon treatment with beta-glucans might contribute to tissue damage of host cells in mucocutaneous candidiasis^44–46^. Consequently curdlan treated Dex-DCs had a reduced ability to induce proliferation, IFN-γ secretion or expression of CD69 and HLA-DR by allogeneic T-Cells.

Although neutrophils are believed to be the major effector immune cells in defense against systemic fungal infection, we believe that impairment of dendritic cell function affects adaptive immune response, that is believed to be important for control of mucosal fungal infection mainly via Th17 cells which are dependent on cytokines like IL-23 and IL1-beta^47–49^. Additionally IL-17 produced by Th17 cells is essential for initial recruitment of neutrophils to the site of infection^50^.

The finding, that SHP-1-expression is lower in Dex-DC might contribute to increased Syk phosphorylation and also STAT3 activation. SHP-1 was shown to be a regulator of STAT3 mediated signaling pathways and is currently under investigation as a target for cancer therapy^51^. Another phosphatase, CD45, which is associated with Dectin-1 signaling, was not differentially expressed in iDC and Dex-DC. Furthermore SHP-1 was shown to be sensitive to oxidative inactivation by ROS^52, 53^, which might contribute to further downregulation of phosphatase-activity in Dex-DC.

As already stated Raf-1 is also involved in Dectin-1-mediated signaling. Raf-1 is thought to shape helper cell differentiation by modulation of the NF-κB proteins. Gringhuis *et al*.^20^ described that Raf-1 activation after stimulation of Dectin-1 with curdlan or *candida albicans* directly influences NF-κB mediated expression of different cytokines. They showed that inhibition or knockout of Raf-1 resulted in abrogated or decreased expression of IL-10, IL-12p35, IL-6 and IL-1beta. On the other hand expression of IL-23 was increased. In our experiments we noticed increased IL-10 production by Dex-DC compared to iDC whereas production of IL-12p70, TNF-alpha and IL-23 was abrogated or decreased. In summary, in our study we identified several mechanisms that promote and support fungal infection upon dexamethasone treatment. Dexamethasone inhibited the function and differentiation of monocyte derived DC and abrogated the immunological effects induced by interaction of fungal beta-glucans with Dectin-1. It inhibited the secretion of cytokines by antigen-presenting cells such as TNF-alpha, IL-12 and IL-23, which are important for T-cell activation and reduced the upregulation of costimulatory molecules on the cell surface upon interaction with beta-glucans. This is probably due to a diminished nuclear expression of several transcription factors involved in DC differentiation and function. Furthermore, dexamethasone increased the expression of Dectin-1 and Syk-phosphorylation but redirected downstream signaling towards STAT-3 that results in production of IL-10, which further contributes to the inhibition of anti-fungal immune responses.

## Material and Methods

### Ethics Statement

Buffy coats for human monocyte isolation were obtained from voluntary blood donors of the University Hospital Bonn. Approval was obtained from the Institutional Ethics Committee of the University of Bonn, North-Rhine Westphalia, Germany (grant number 173/09).

Written informed consent was obtained for blood donation and further processing of blood samples for scientific purposes by the blood bank/transfusion medicine of the University Hospital of Bonn.

All experiments were performed in accordance with relevant guidelines and regulations.

### Media and reagents

Cells were cultured in RPMI 1640 containing glutamax-I, supplemented with 10% inactivated fetal calf serum (RP10 medium) and 1% penicillin/streptomycin (Invitrogen). All reagents not otherwise indicated were purchased from Sigma-Aldrich. HKCA were purchased from InvivoGen (San Diego, CA). Curdlan was purchased from Wako Chemicals USA, Inc. (Richmond, Virginia). Zymosan A was purchased from Sigma-Aldrich.

### Detection of superoxide-anion generation

Cells were washed and plated in 24-well cell-culture plate at 5 × 10^5^ per well in fresh RP10 medium and were allowed to settle for 2–4 h. Cells were incubated with Inhibitors at the indicated concentrations or an anti-hDectin-1 antibody (R&D Systems, 5 µg/ml) 30 min prior to stimulation with the beta-glucans zymosan (100 µg/ml) or curdlan (100–400 µg/ml). At timepoint of stimulation the superoxide detection reagent (Enzo Life Sciences) was applied to the cells. Cells were stimulated for 45 min, harvested and analyzed by flow cytometry.

### Generation of iDCs and Dex-DCs

Human monocyte-derived DCs (iDCs) were generated from peripheral blood by plastic adherence as described previously^10, 54^. Adherent monocytes were cultured in RP10 medium supplemented with GM-CSF (100 ng/mL; Leukine, Liquid Sargramostim) and IL-4 (20 ng/ml; R&D Systems). Cytokines were added to the cells every other day.

To generate Dex-DC adherent monocytes were treated as described for iDC. Dexamethasone was added furhter at 100 nM every other day. In each case, equal amounts of EtOH were added as a control.

### Mixed lymphocyte reaction

1 × 10^4^ of iDCs or Dex-DCs were cultured with a total of 1 × 10^5^ responding allogeneic PBMNC as described previously^54^. Tritium-labeled thymidine incorporation was measured on day 5 by a 16-hour pulse with [^3^H]-thymidine (18.5 kBq/well; GE Healthcare).

### Immunostaining for flow cytometry

iDC and Dex-DC were harvested, washed and stained using commercially available mAbs from BD Biosciences, Beckmann Coulter and R&D Systems. Cells were in some cases stimulated for 18 h with zymosan or curdlan (both at 100 µg/ml) prior to flow cytometric analysis. Flow cytometry data was analyzed using FlowJo.

### Determination of cytokine production

Concentration of cytokines in cell-culture supernatants was determined using DuoSet® ELISA Development Systems (R&D systems) according to the manufacturer’s instructions. Cells were harvested, washed and seeded in 24-well plates in fresh RP10 media prior to stimulation. Dectin-1 stimulation was accomplished as described for flow cytometric experiments. Inhibitors were added to the cells 30 min prior to stimulation at the indicated concentrations.

### Polyacrylamide gel electrophoresis and Western blotting

Whole cell lysates were prepared as described previously^54^. Protein concentrations were determined using a bicinchoninic acid assay (Pierce, Perbio Science). For analysis of nuclear protein out of 10^6^ cells, 20 μl of nuclear extracts were separated on a 10% SDS-PAGE and transferred on a nitrocellulose membrane. Ponceau S staining of the membrane was performed to ensure that equal amounts of protein had been loaded onto the gel. Subsequently, the blot was probed with the indicated antibodies. Protein bands were detected using an enhanced chemiluminescence kit (GE Healthcare).

### Statistical analysis

All experiments were performed at least 3 times, with representative experiments shown. To analyze statistical significance, a Student’s t test was used.

## Electronic supplementary material

Supplementary Information

## References

[1] Lionakis MS, Kontoyiannis DP (2003). Glucocorticoids and invasive fungal infections. The Lancet.

[2] Liao J (2014). Dexamethasone potentiates myeloid-derived suppressor cell function in prolonging allograft survival through nitric oxide. J Leukoc Biol.

[3] Unger WWJ, Laban S, Kleijwegt FS, van der Slik AR, Roep BO (2009). Induction of Treg by monocyte-derived DC modulated by vitamin D3 or dexamethasone: differential role for PD-L1. Eur J Immunol.

[4] Xia C-Q, Peng R, Beato F, Clare-Salzler MJ (2005). Dexamethasone induces IL-10-producing monocyte-derived dendritic cells with durable immaturity. Scand J Immunol.

[5] Brown GD (2002). Dectin-1 is a major beta-glucan receptor on macrophages. J Exp Med.

[6] Brown GD (2003). Dectin-1 mediates the biological effects of beta-glucans. J Exp Med.

[7] Kankkunen P (2010). (1,3)-beta-glucans activate both dectin-1 and NLRP3 inflammasome in human macrophages. J Immunol.

[8] del Fresno C (2013). Interferon-beta production via Dectin-1-Syk-IRF5 signaling in dendritic cells is crucial for immunity to C. albicans. Immunity.

[9] Goodridge HS, Simmons RM, Underhill DM (2007). Dectin-1 stimulation by Candida albicans yeast or zymosan triggers NFAT activation in macrophages and dendritic cells. J Immunol.

[10] Kock G (2011). Regulation of dectin-1-mediated dendritic cell activation by peroxisome proliferator-activated receptor-gamma ligand troglitazone. Blood.

[11] Rogers NC (2005). Syk-dependent cytokine induction by Dectin-1 reveals a novel pattern recognition pathway for C type lectins. Immunity.

[12] Carvalho A (2012). Dectin-1 isoforms contribute to distinct Th1/Th17 cell activation in mucosal candidiasis. Cell Mol Immunol.

[13] Dillon S (2006). Yeast zymosan, a stimulus for TLR2 and dectin-1, induces regulatory antigen-presenting cells and immunological tolerance. J Clin Invest.

[14] Eberle ME, Dalpke AH (2012). Dectin-1 stimulation induces suppressor of cytokine signaling 1, thereby modulating TLR signaling and T cell responses. J Immunol.

[15] Agrawal S, Gupta S, Agrawal A (2010). Human Dendritic Cells Activated via Dectin-1 Are Efficient at Priming Th17, Cytotoxic CD8 T and B Cell Responses. PLOS ONE.

[16] Weck MM (2008). hDectin-1 is involved in uptake and cross-presentation of cellular antigens. Blood.

[17] Leibundgut-Landmann S, Osorio F, Brown GD (2008). & Reis e Sousa, C. Stimulation of dendritic cells via the dectin-1/Syk pathway allows priming of cytotoxic T-cell responses. Blood.

[18] Underhill DM, Rossnagle E, Lowell CA, Simmons RM (2005). Dectin-1 activates Syk tyrosine kinase in a dynamic subset of macrophages for reactive oxygen production. Blood.

[19] Gringhuis SI (2012). Dectin-1 is an extracellular pathogen sensor for the induction and processing of IL-1 beta via a noncanonical caspase-8 inflammasome. Nat Immunol.

[20] Gringhuis SI (2009). Dectin-1 directs T helper cell differentiation by controlling noncanonical NF-kappaB activation through Raf-1 and Syk. Nat. Immunol..

[21] Ferwerda B (2009). Human Dectin-1 Deficiency and Mucocutaneous Fungal Infections. N. Engl. J. Med..

[22] Zelante T (2007). IL-23 and the Th17 pathway promote inflammation and impair antifungal immune resistance. Eur J Immunol.

[23] Ganesan S (2014). Caspase-8 modulates Dectin-1 and CR3 driven IL-1β production in response to β-glucans and the fungal pathogen, Candida albicans1. J. Immunol. Baltim. Md 1950.

[24] Cambi A (2003). The C-type lectin DC-SIGN (CD209) is an antigen-uptake receptor for Candida albicans on dendritic cells. Eur. J. Immunol..

[25] Ferwerda G, Meyer-Wentrup F, Kullberg B-J, Netea MG, Adema GJ (2008). Dectin-1 synergizes with TLR2 and TLR4 for cytokine production in human primary monocytes and macrophages. Cell. Microbiol.

[26] Gross O (2009). Syk kinase signalling couples to the Nlrp3 inflammasome for anti-fungal host defence. Nature.

[27] Thiagarajan PS (2013). Vimentin is an endogenous ligand for the pattern recognition receptor Dectin-1. Cardiovasc Res.

[28] Jia X-M (2014). CARD9 mediates Dectin-1-induced ERK activation by linking Ras-GRF1 to H-Ras for antifungal immunity. J Exp Med.

[29] Melillo JA (2010). Dendritic Cell (DC)-Specific Targeting Reveals Stat3 as a Negative Regulator of DC Function. J. Immunol..

[30] Yu H, Kortylewski M, Pardoll D (2007). Crosstalk between cancer and immune cells: role of STAT3 in the tumour microenvironment. Nat. Rev. Immunol..

[31] Tong X, Yin L, Washington R, Rosenberg DW, Giardina C (2004). The p50-p50 NF-kappaB complex as a stimulus-specific repressor of gene activation. Mol. Cell. Biochem..

[32] Cao S, Zhang X, Edwards JP, Mosser DM (2006). NF-kappaB1 (p50) homodimers differentially regulate pro- and anti-inflammatory cytokines in macrophages. J. Biol. Chem..

[33] Conner JR, Smirnova II, Moseman AP, Poltorak A (2010). IRAK1BP1 inhibits inflammation by promoting nuclear translocation of NF-κB p50. Proc. Natl. Acad. Sci.

[34] Goodridge HS (2011). Activation of the innate immune receptor Dectin-1 upon formation of a ‘phagocytic synapse’. Nature.

[35] Alsadeq A, Hobeika E, Medgyesi D, Kläsener K, Reth M (2014). The role of the Syk/Shp-1 kinase-phosphatase equilibrium in B cell development and signaling. J Immunol.

[36] Huang Z-Y, Hunter S, Kim M-K, Indik ZK, Schreiber AD (2003). The effect of phosphatases SHP-1 and SHIP-1 on signaling by the ITIM- and ITAM-containing Fcgamma receptors FcgammaRIIB and FcgammaRIIA. J Leukoc Biol.

[37] Kant AM (2002). SHP-1 regulates Fcgamma receptor-mediated phagocytosis and the activation of RAC. Blood.

[38] Hernández-Santos N, Gaffen SL (2012). Th17 cells in immunity to Candida albicans. Cell Host Microbe.

[39] Rescigno M, Martino M, Sutherland CL, Gold MR, Ricciardi-Castagnoli P (1998). Dendritic Cell Survival and Maturation Are Regulated by Different Signaling Pathways. J. Exp. Med..

[40] Grumont R (2001). C-Rel Regulates Interleukin 12 P70 Expression in Cd8+ Dendritic Cells by Specifically Inducing p35 Gene Transcription. J. Exp. Med..

[41] Carotta S (2010). The transcription factor PU.1 controls dendritic cell development and Flt3 cytokine receptor expression in a dose-dependent manner. Immunity.

[42] Nakahara T (2004). Role of c-Jun N-terminal kinase on lipopolysaccharide induced maturation of human monocyte-derived dendritic cells. Int. Immunol..

[43] Kawai T, Akira S (2006). TLR signaling. Cell Death Differ.

[44] Fratti RA, Belanger PH, Ghannoum MA, Edwards JE (1998). & Filler, S. G. Endothelial Cell Injury Caused by Candida albicans Is Dependent on Iron. Infect. Immun..

[45] Ricevuti G (1997). Host tissue damage by phagocytes. Ann. N. Y. Acad. Sci.

[46] Mittal M, Siddiqui MR, Tran K, Reddy SP, Malik AB (2014). Reactive Oxygen Species in Inflammation and Tissue Injury. Antioxid. Redox Signal..

[47] Netea MG (2010). IL-1beta processing in host defense: beyond the inflammasomes. PLoS Pathog.

[48] Engelhardt KR, Grimbacher B (2012). Mendelian traits causing susceptibility to mucocutaneous fungal infections in human subjects. J. Allergy Clin. Immunol..

[49] Puel A (2011). Chronic Mucocutaneous Candidiasis in Humans with Inborn Errors of Interleukin-17 Immunity. Science.

[50] Kashem SW, Kaplan DH (2016). Skin Immunity to Candida albicans. Trends Immunol..

[51] Fan L-C (2015). SHP-1 is a negative regulator of epithelial–mesenchymal transition in hepatocellular carcinoma. Oncogene.

[52] Ke K (2014). Reactive oxygen species induce the association of SHP-1 with c-Src and the oxidation of both to enhance osteoclast survival. Am. J. Physiol. Endocrinol. Metab..

[53] Meng T-C, Fukada T, Tonks NK (2002). Reversible oxidation and inactivation of protein tyrosine phosphatases *in vivo*. Mol Cell.

[54] Heine A (2013). The JAK-inhibitor ruxolitinib impairs dendritic cell function *in vitro* and *in vivo*. Blood.

