# Supplementary material for: Dexamethasone induced inhibition of Dectin-1 activation of antigen presenting cells is mediated via STAT-3 and NF-κB signaling pathways
Source: Sci Rep. 2017 Jul 3;7:4522. doi: 10.1038/s41598-017-04558-z (PMC5495798; doi:10.1038/s41598-017-04558-z)

Dexamethasone induced inhibition of Dectin-1 activation of antigen presenting cells is mediated via STAT-3 and NF-κB signaling pathways.

Philipp Kotthoff[1]*, Annkristin Heine[1], Stefanie Andrea Erika Held[1] and Peter Brossart[1]

[1]Medical Clinic III for Oncology, Hematology, Immuno-Oncology and Rheumatology, University Hospital Bonn

Sigmund-Freud-Straße 25, 53127 Bonn, Germany

Tel: 0049228/287-22234, Fax: 0049228/287-22635

Supplementary Figures

Figure S1

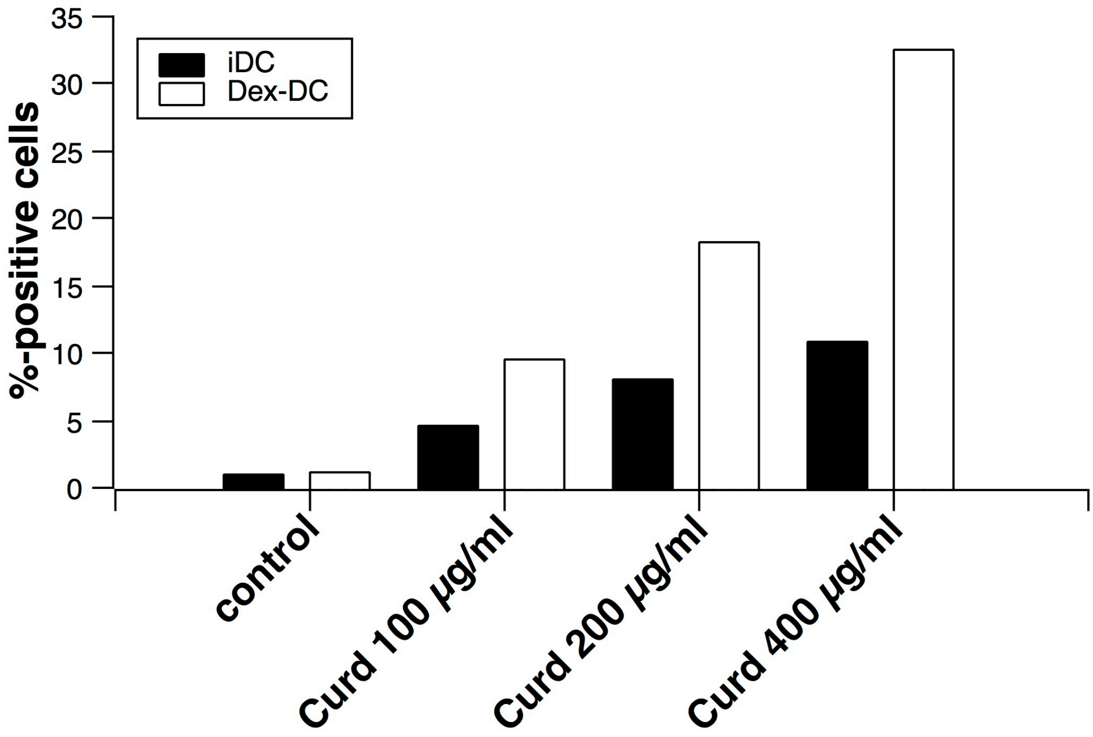

Figure S2

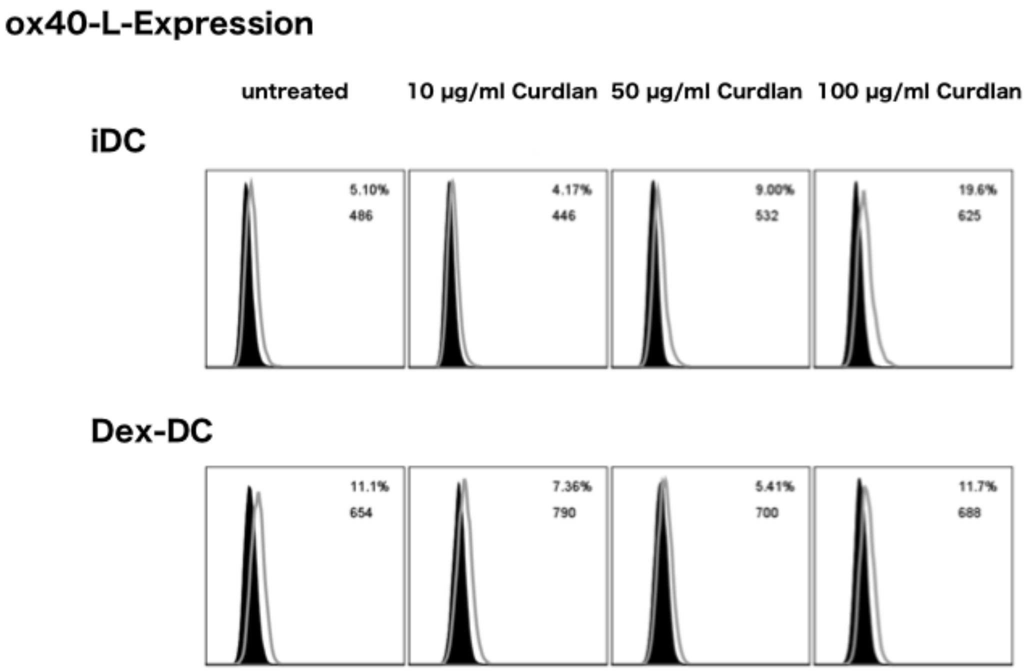

ox40-L-Expression
untreated
10 µg/ml Curdlan
50 µg/ml Curdlan
100 µg/ml Curdlan
iDC
5.10%
486
4.17%
446
9.00%
532
19.6%
625
Dex-DC
11.1%
654
7.36%
790
5.41%
700
11.7%
688

Figure S3

Dex-DC ± 100 µg/ml Curdlan

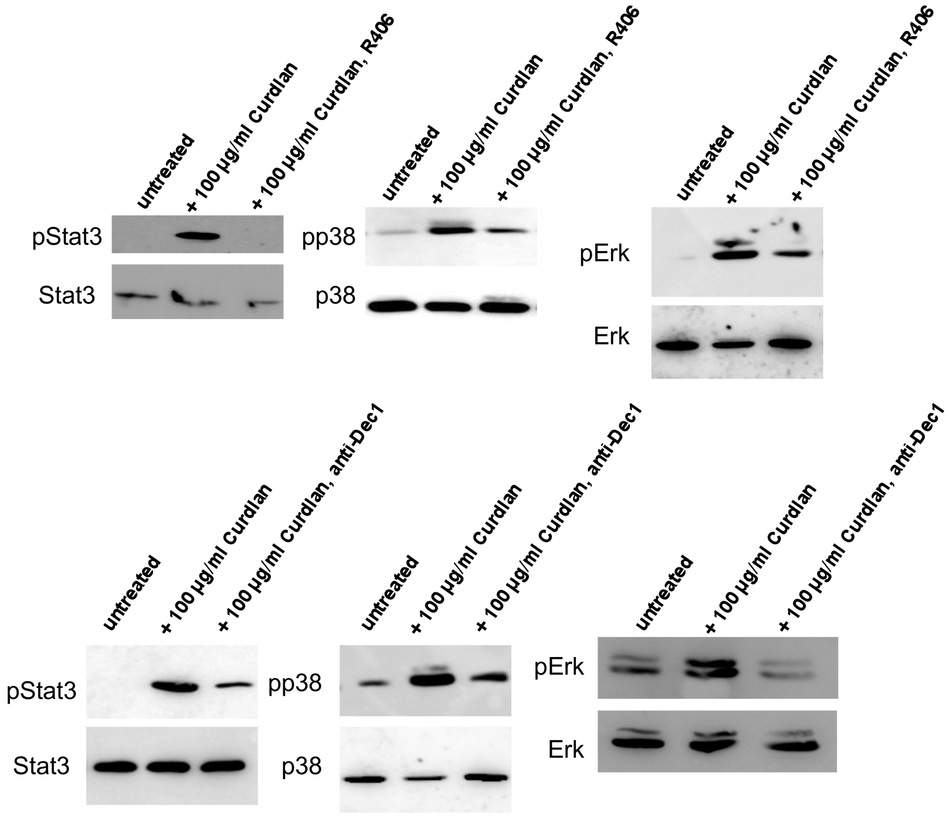

Figure S1. Curdlan induced superoxide-anion generation.

iDC and Dex-DC were treated for 45 min with increasing concentrations of curdlan (0 – 400 μg/ml) and generation of superoxide-anions was analyzed by flow cytometry. Superoxide-anion detection reagent was used 1:5000 and was added to cells at time point of stimulation.

Figure S2. ox40-L-expression on Dex-DC and iDC.

iDC and Dex-DC were treated for 24 hours with increasing concentrations of curdlan (0 – 100 μg/ml) and expression of ox40-L was analyzed by flow cytometry as described.

Figure S3. Curdlan induced phosphorylation of p38, Stat3 and Erk is reduced by anti-Dectin-1 specific antibody and Syk Inhibitor

iDC or Dex-DC were treated for 30 min with 100 μg/ml curdlan. Lysates were separated by SDS-PAGE and were tested for phosphorylated p38, Erk and Stat3. Pretreatment of iDC or Dex-Dc with R406 (5 μM) oder anti-Dectin-1 antibody (10 μg/ml) for 30 min suppressed phosphorylation of Erk, p38 and Stat3 after incubation with curdlan.

Full Western-Blots for Figure 4 and 6

Figure 4

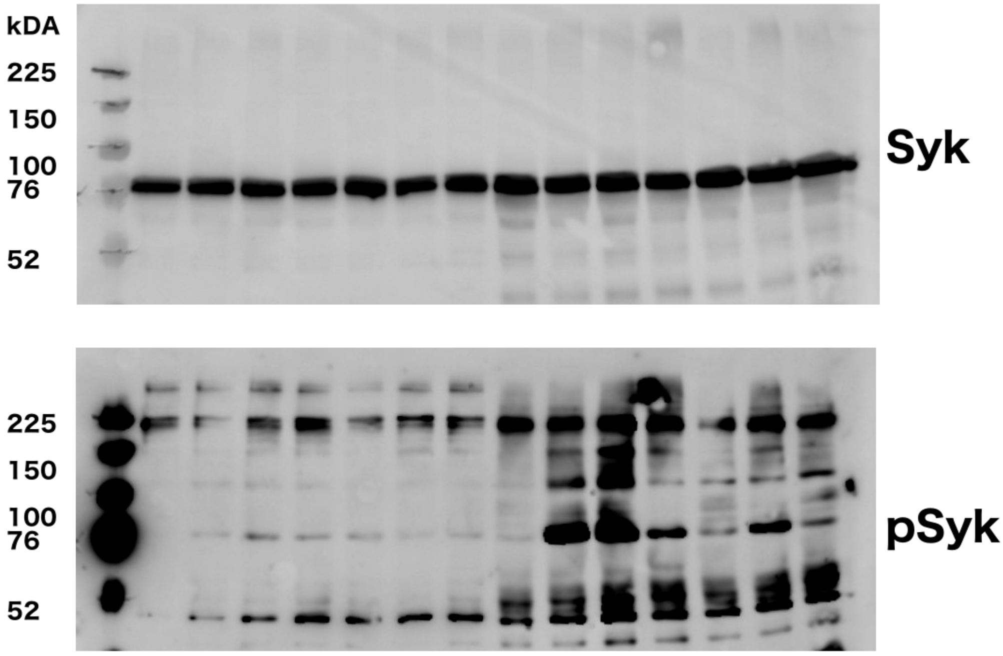

Figure 6 C

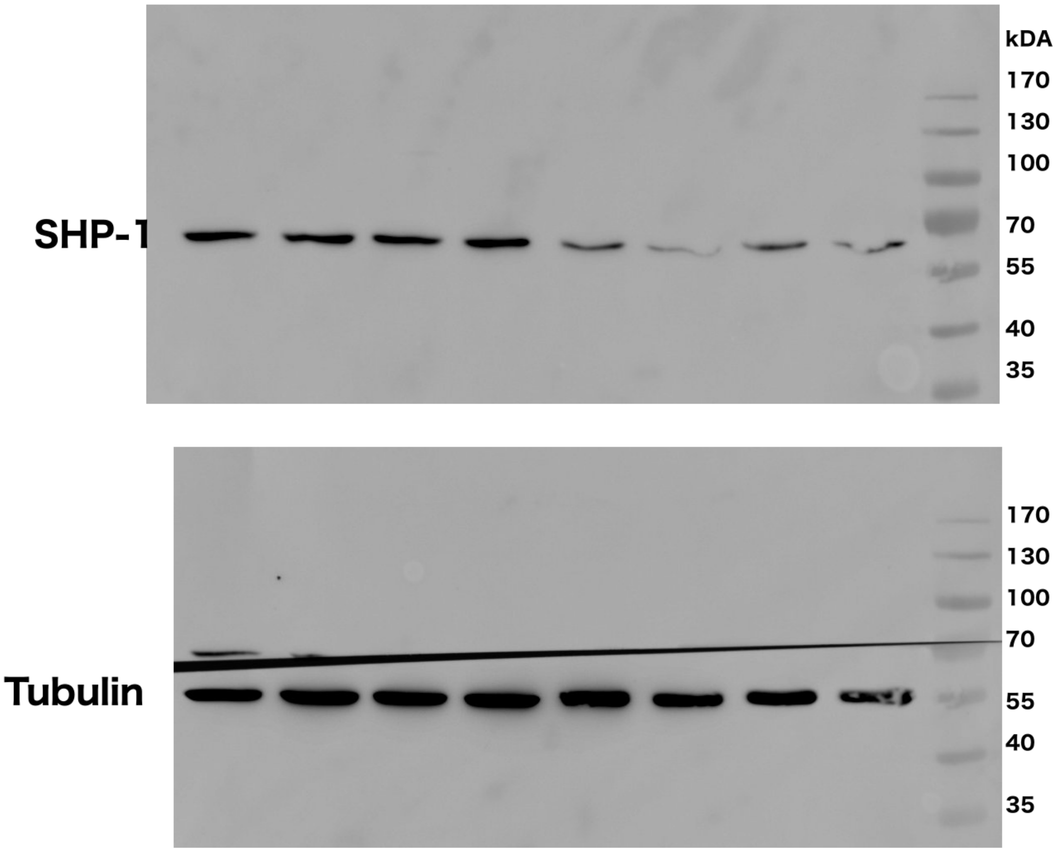

Supplement: Supplementary file 1 — Supplementary Information [file 41598_2017_4558_MOESM1_ESM.pdf]
